# Cholera Infantum

**Published:** 1862-09

**Authors:** W. Owen Brown

**Affiliations:** Providence


### CHOLERA INFANTUM.

By W. OWEN BROWN, M. D., of Providence.

*Read at the Quarterly Meeting of the Rhode Island Medical Society, Oct.* 2, 1861

This disease occurs in New England in the hot weather of summer and early autumn, and is so familiar to most physicians, as to require no very minute definition. It is characterized by diarrhœa, vomiting, and great prostration of the system. The discharges from the bowels are variously colored, as green, dark brown, or resembling rice water. The odor is often very fetid, similar to that of putrescent meat, arising, perhaps, from the absence of the antiseptic influence of healthy bile, in due amount, in the intestinal canal. The fever which is usually present, occasionally assumes a remittent type. The bills of mortality in most cities, and the experience of physicians in the localities where it prevails, testify to its fearful fatality.

History.—It is doubted if there is another disease, which annually destroys so many lives, about which so little has been written. Authorities tell us that in our large cities, during the summer months, about one-fourth of all the deaths, among children, arise from this disease. During the year 1860, five hundred and fourteen children are reported as having died from it in Philadelphia alone, which is perhaps not more than the average mortality from this cause, in most of the large cities in this country.

Though it causes this frightful destruction of infant life every year, yet the name of Cholera Infantum, or its synonyms, is not found in the indices to Braithwaite's Summary of Medicine and Surgery, during the six years from 1854 to 1860, thus leaving the inference that not an important article was written upon this subject during all that time. In the Year Book of Medicine and Surgery, of the Sydenham Society, for 1859, it is true that two articles upon the Pathological Appearances in Cholera Infantum are alluded to; both were published in that year. In one of these articles, the writer states that in many cases, the sinuses of the dura mater were found "filled with fresh blood coagula, and with fibrinous formations more or less adherent and firm," and hence arrives at the conclusion that the rapid loss of water from the blood, favors the formation of venous thrombus in the brain, and thus occasions sudden death, in many cases. In the other paper alluded to, the author relates four cases, in all of which he found inflammation of the colon, which he considers a constant attendant upon the disease. Besides these articles, the Year Book alludes to a paper upon Cholera Infantum, in the United States, as published in New York in 1858, and also to a paper published in the Gazette des Hopitaux, in 1858, entitled Weaning, —its Relations to Cholera Infantum. Pethaps the most valuable article yet written upon Cholera Infantum, is by Dr. Jas. Stewart, published in New York, in 1857. This paper, however, is not intended to present a biblography of this subject. In the various medical periodicals, there are remarks upon the "Diarrhœa of Infants," which, from the kindred nature of the diseases, affords much valuable information relating to the subject immediately before us.

Dr. Condie states that "the disease occurs as an epidemic in all the large cities, throughout the middle, the southern, and most of the western States, during the season of the greatest heats, making its appearance and ceasing, earlier or later, according as the summer varies in the period of its commencement and close." "In the more southern States, it appears as early as April or May, and frequently cases of it occur until late in September."

Dr. Copland remarks, "that in some very nnhealthy climates within the tropics, the children born of European parents seldom reach two or three years, without having an attack, and in some places scarcely one will survive this age, if suffered to remain in them. It is certainly not an infrequent malady among children in the English metropolis."

Symptoms.—The disease, like Cholera, is often preceded by diarrhœa, but not unfrequently it commences at once, like cholera morbus, with vomiting and purging, and death sometimes occurs within twenty-four hours. More commonly it comes on with frequent diarrhœa, attended by occasional vomiting, with marked pyrexia, and the case may be protracted from one to several weeks, ending in complete irritability of the stomach, collapse, and, perhaps, cerebral effusions.

The diarrhœa may linger for months, even, and with the approach of cold weather, yield to treatment, or the recuperative powers of the system may then, alone, prove adequate to recovery.

Pathology.—This has often been minutely and ably described, and it is not the intention of the writer to go into detail, nor could he state anything original upon this head.

In cases of an early death from an attack, it is known that the mucus membrane of the alimentary canal is unusually pale, the liver frequently congested,—sometimes enlarged, and observers are perhaps unanimous in the statement that the *mucous follicles* of the alimentary canal are enlarged. Dr. Horner describes the appearance of the enlarged follicles as "resembling a sprinkling of white sand upon the surface of the mucous membrane."

In more protracted cases, the mucous membrane of the stomach and intestines exhibits evidence of inflammation in red points, and the glandular follicles are enlarged. Dr. Horner "found the mucous follicles enlarged, and even ulcerated, both in the small and large intestines, and in one case, ulcerations of the surface of the membrane in the jejunum.

Pathologists appear to be united in the opinion that there is present, *at first*, merely an *enlarged* condition of the *mucous follicles*, with irritation of the mucous membrane, and congestion and enlargement of the liver. At a more advanced stage, inflammation of the gastro-enteric mucous membrane, with, sometimes, ulceration and softening.

At almost any period of the attack, the meninges of the brain are liable to be involved, and serous, or sero-sanguineous, effusion to take place.

Causes.—The disease is known to be almost wholly confined to children under two years of age. The *mucous follicles* of such are "liable to be *largely developed*," and the mucous membrane is peculiarly sensitive. During dentition, the buccal mucous membrane, as well as the salivary glands, perform their functions of secretion and excretion with unusual activity; by reflex action, the nervous irritability produced by the pressure of the advancing teeth against the gums, or against the nervous filliments distributed to the gums, would excite the mucous membrane of the stomach and intestines to morbid action; any indigestible substances taken as food, may increase this action, and the relaxing and debilitating effect of the hot, close, damp air of cholera infantum localities, together with the unascertained atmospheric poison absorbed into the system from such localities, complete the conditions essential to the production of this fatal disease.

It is well known that during the heat of summer, certain sudden changes, as from dry and warm to damp, sultry, "close" weather, will give origin to numerous cases of cholera morbus in adults; it is believed, that in large cities, corresponding atmospheric changes are attended by a large increase of cases of cholera infantum among children,—that causes which would, in adults, have produced cholera morbus, in the more sensitive and impressible digestive organs of the child, induce cholera infantum.

Nature.—The pathological conditions throw much light upon the nature of this affection, rendering it very safe to regard it as essentially, at first, a *gastro enteric irritation*, passing into gastro-enteritis, with the concomitant lesions, involving especially the mucous follicles of the alimentary canal, complicated with congestion and enlargement of the liver; and made much more virulent by a depraved condition of the blood, induced by atmospheric toxæmia.

Treatment.—At the onset of an attack, the indications, reasoning from the pathology and nature of the disease, are:

1st. To quiet nervous irritability, whether arising from dentition, or other cause affecting any part of the digestive organs, and which, by reflex action, may induce vomiting or diarrhœa.

2d. To arrest the increased secretion and excretion of the mucous follicles, and thereby arrest another source of diarrhœa and vomiting.

3d. To control the fever induced by the blood poison, and give the system an opportunity to recover itself.

4th. At a more advanced stage to guard against collapse, or to arouse the system from it when it has supervened.

5th. The treatment of the sequelæ.

To meet these indications, the following remedies, variously combined, have been found very efficient:

Opium, or its preparations; cresote, calomel, and mercury with chalk; aromatics, as aromatic syrup of rhubard, infusion of cinnamon, or of spearmint; astringents, as rhatany, in some of its preparations, and acetate of lead; to these may be added quinia, iron, mineral acids and ice.

To a child under two years, one twenty-fourth to one sixteenth of a grain of powdered opium, may be given, with half a grain to a grain of calomel, to promote the action of the liver; or, where there is not much gastric irritation, one to two grains of Dover's Powder, with a grain of mercurialized chalk, may fulfill the first indication to quiet nervous irritation.

The addition of a grain of acetate of lead, to either of the above, restrains the action of the secretory surface of the intestines, or constringes the mucous follicles, and at the same time, perhaps, allays febrile excitement.

Creosote is known to have a powerful effect in restraining serous discharges, and it seems highly probable that its antiseptic properties may be of advantage, since the discharges are so often highly putrescent; besides, it is often useful in allaying gastric irritation.

Aromatics, acting as local stimulants of the digestive organs, often prevent or overcome the want of tone which gives origin to morbid excretion.

Any successful attempt at arresting the diarrhœa and vomiting, is liable to result in an increase of fever, with great thirst; the profuse discharges from the stomach and bowels deprives the blood of its watery part, and in this manner, thirst is also induced.

Ice, given in small quantities, at frequent intervals, most happily controls the fever, and allays the thirst.

Since the preparation of this paper was commenced, the writer has had under treatment a child of some twenty months, sick with cholera infantum, which lay, part of the time, in a semi-unconscious condition, but was disturbed at intervals by pain, or aroused by thirst, and then so eager for fluids as to take nearly indifferently, unpalatable medicines or grateful drinks. When a lump of ice was placed in this child's mouth, with the first cooling sensation, it awoke from its lethargy, and its face fairly beamed with pleasure. From that moment it began to amend, and recovered from a nearly hopeless condition.

Dr. Mauran, of this city, relates an instance which occurred to him many years ago, when ice was not so much used in practice as now. He was called to a child nearly in the collapsed stage of cholera infantum. It was vomiting most profusely a dark substance resembling coffee grounds. It entreated water from every one who approached, and vomited it instantly. Dr. M. called for some ice, and a lump was placed in the child's mouth, and retained with great satisfaction. Regarding the case as hopeless, and seeing the comfort the ice afforded, the Doctor sat by and gave it piece after piece, until, in a very brief time, the child had swallowed an entire saucer full. The ice was continued during the night as the child desired it. This patient rapidly recovered.

The fourth condition, or tendency to collapse, or collapse itself, is, of course, best met with hot applications, cordials and stimulants, if the stomach will bear them; but too often little remains which can be done with much hope of recovery.

In the Transactions of the Medical Society of the State of New York, for 1861, is an article upon "Dermoid Medication," which, it is said, "has often proved successful, in cases of cholera infantum, when all other means had failed." The skin is first cleansed with soap and warm water, and excited by frictions. Then a flannel, sufficiently large to cover the abdomen, is saturated with a solution of one scruple of corrosive sublimate, in six fluid ounces of alcohol. The flannel is sprinkled with laudanum and laid upon the bowels; over this is placed another layer, saturated with a hot infusion of spices. The whole is covered with oiled silk, and suffered to remain from twenty-four to forty-eight hours. Ice, iced champaigne, &c., to be given internally.

The *sequelæ* are most likely to be chronic diarrhœa, with anæmia, or marasmus. For the treatment of these affections, quinia, or quinia and iron, seem most admirably adapted, as the following:

℞. Quiniæ et Ferri Citratis.................. ʒ ij.

Syrupi Zingiberis...................... f. ℥ iv.

Tincturæ Opii......................... f. ʒ ss.

*Misce.*—Dose, half a teaspoonful once in four hours, to a child under two years old.

It is familiar to the profession, that after opiates have failed to restrain a diarrhœa, quinia, or quinia and iron, will most effectually accomplish this end. In an able article in the *American Journal of Medical Sciences*, No. LXXXIII, N. S., p 53, the writer says of "the more obvious and important effects of quinia, none is of so much importance as its power of giving contractile action to the capillaries. This property of quinia gives it a power over almost all forms of venous and capillary congestion, which, perhaps, it is impossible to obtain by any other known agent." In concluding his remarks upon the known antagonistic effects of opium and quinia, he says, "he is forced to the conclusion, that they are not so far antagonistic that they should never be administered together."—*Ibid. p.* 58.

Since the above was written, the writer has met with the following extracts, from the Transactions of the Illinois State Medical Society, for 1860:

Speaking of the Diarrhæa of Children, Dr. J. O. Harris says:—"In this disease I find that after exhausting all the usual remedies advised by our standard authors, and by my brother physicians, that quinine, in *full* doses, frequently repeated, acted (or seemed to act) admirably. I thought at the time that I was prescribing empirically, and now do not pretend to explain the modus operandi of the remedy. I only know this, that my patient recovered under the use of quinine, and I still prescribe it, when I see no particular indication for its use."

Dr. J. S. Rich, of Florida, in an article upon Cholera Infantum, says he was remarkably successful in treating it with large doses of quinine. The following was given to his own child:

℞. Quiniæ Sulphatis....................grs. v.
Hydrargyri Chloridi Mitis............grs. iij.
Olei Terebinthinæ...................gtt. xx.
Mellis.............................f. ℈ j.

*Misce.*—Give the whole at a dose.

After about twelve hours, the same dose was repeated, omitting the calomel. He says, "I could not detect any other effect of the quinine than a most profound and salutary sleep; the pulse, which had been much too frequent, irregular, thread-like and fluctuating, became slow as in health, and firm; the skin was continually moist, with warm perspiration; the kidneys acted most copiously; the bowels acted twice, but the last showing that the liver was performing its healthy functions. The most remarkable change was the disappearance of the general cadaverous aspect and *grave-yard odor.* The return of appetite, and general powers of digestion, was extraordinary."—*Philadelphia Medical and Surgical Journal, May* 24, 1862.

The following is a summary of treatment which the writer has found happily to fulfill many of the conditions suggested in this paper.

In the early stages, the following mixture is given, unless there are manifest contra-indications, including all the ingredients, or omitting those not apwarently required:

℞. Syrupi Acaciæ........................f. ℥ iss.

Syrupi Rhei Aromatici..............f. ℥ ss.

Syrupi Krameriæ....................... ℥ ss.

Spiritus Ammoniæ Aromatici..........f. ʒ j.

Tincturæ Oppi.........................f. ʒ ss.

Creasoti...............................gtt. iv–vi.

*Misce.*—To a child of one year or under, give half a teaspoonful once in four hours, gradually increasing the dose to a teaspoonful.

If this does not check the discharges, give at alternate doses, one twenty-fourth to one sixteenth of a grain of powdered opium, with half a grain of calomel, or omitting the first preparation, give the last once in four hours. If still more restraining effect is desired, add to the last half a grain of acetate of lead.

At any stage of the disease, if there is evidence of acidity in the primæ viæ, one or two grains of the bicarbonate of soda are added to the above powder, of course omitting the acetate of lead, when bicarbonates are given. Ice, as has been before suggested, or infusion of spearmint, cold, when there is not much gastric irritability. If there is much tenderness of the abdomen, mustard cataplasms, carefully guarding against vesication, or cloths wet with mustard and vinegar, pepper sauce, or alcohol, are applied to the abdomen. When there are are head symptoms, mustard cataplasms to the feet.

Dr. Condie states that minute doses of calomel rubbed up with sugar, and placed upon the tongue, will usually allay the vomiting.

Of course this treatment is not in any manner intended to suspend the paramount advantages to be derived from pure air, or a change of air, when practicable.

